# The largest and earliest known sample of dental caries in an extinct mammal (Mammalia, Euarchonta, *Microsyops latidens*) and its ecological implications

**DOI:** 10.1038/s41598-021-95330-x

**Published:** 2021-09-09

**Authors:** Keegan R. Selig, Mary T. Silcox

**Affiliations:** Department of Anthropology, University of Toronto Scarborough, Toronto, ON Canada

**Keywords:** Anthropology, Palaeontology

## Abstract

Dental cavities or caries is a common disease among modern humans, affecting almost every adult. Caries frequency has been used to study dietary change in humans over time, based on an inferred tie between the incidence of caries and a carbohydrate-rich diet. However, the disease is not unique to our species. Among non-human primates, there is also variation in caries frequency associated with diet, suggesting that this metric may provide a mechanism for studying diet in broader contexts, and across geological time. To date, very few studies have examined caries among fossil mammals, and none have done so among Eocene mammals. Here, we present our analysis of the largest sample to date of fossil caries in a single extinct mammal species, *Microsyops latidens*, a stem primate from the early Eocene, which is known from over a thousand specimens from the Southern Bighorn Basin of Wyoming (n = 1030). Our results show that *Microsyops latidens* is characterized by a high prevalence of dental caries (7.48% of individuals), with notable variation through time, reaching 17.24% of individuals from a particular interval. This interval is also associated with a change in overall dental form, as quantified by dental topographic analysis, which measures functional aspects of the chewing surface of teeth. These observations suggest that this species experienced a shift in their diet to include more fruit or other sugar rich-foods for a short period. Our analysis, therefore, suggests that the diet of *M. latidens* fluctuated over time, as well as providing a framework for assessing caries in other fossil taxa.

## Introduction

Dental caries is a prevalent disease among humans, affecting nearly every adult during their lifetime^1^. However, caries is not unique to humans, nor is it a recent phenomenon^2–6^. Among the potential factors influencing caries occurrence are salivary and oral biochemistry, dietary abrasiveness, and fissure depth. But perhaps the most common cause of caries occurs when dental tissues are demineralized by oral bacteria releasing acids as they digest carbohydrates in the mouth. As such, a diet high in carbohydrate-rich foods, such as fruits, gums, or honey, has been associated with higher caries frequency^7,8^. Caries, therefore, provides insight into important facets of the life of an individual such as diet and health, so patterns of caries frequencies in the past have the potential to shed light on the ecology of extinct animals. Whereas dental caries can potentially provide useful information for reconstructing the diet of extinct animals e.g.,^9,10^, palaeontologists tend to study dental morphology and dental wear as proxies for diet e.g.,^11–14^. One method for studying dental morphology is a series of methods known as dental topographic analysis (DTA) e.g.,^15–23^. Dental topographic analysis treats the occlusal surface of a tooth like a topographic map, to which several algorithms can be applied to measure functional, three-dimensional aspects of the teeth including curvature (Dirichlet normal energy^24^), complexity (three-dimensional orientation patch count rotated^25,26^) and relief (relief index^16,27,28^).

The etiology of caries has been studied in great depth in humans, and to some degree in non-human primates^2,29^. Studies have also examined caries in a handful of fossil mammals from the Mid-Miocene to the Late Pleistocene, including studies on fossil primates, bears, and artiodactyls^5,6,9,10,30–33^; however, these incidences are rare, with only small samples of individuals showing caries (Table 1). To date, very little is known about caries in older fossil mammals, or about how caries frequency may vary over time within a single species.

**Table 1:** List of fossil mammal species previously examined for the presence of dental caries.

Order	Taxon	Age	Specimens	% Caries	References
Primates	*Microsyops latidens*	Early Eocene	1030 Individuals	7.48%	This study
	*Dryopithecus carinthiacus*	Mid-Miocene	1 Individual	–	Fuss et al.^10^
	*Oreopithecus bambolii*	Late Miocene	1 Individual	–	Rossi et al.^30^
	*?Indopithecus *sp.	Late Miocene	1 Tooth	–	Patniak et al.^31^
	*Early (pre-agricultural) Homo*	Pliocene-Pleistocene	44 Teeth	4.60%	Compiled by Towel et al.^5^
	*Australopithecus africanus*	Mid-Pliocene-Early Pleistocene	328 Teeth	0.00%	Compiled by Towel et al.^5^
	*Gigantopithecus blacki*	Early Pleistocene	62 Teeth	24.29%	Zhao^32^
	*Paranthropus robustus*	Early-Mid-Pleistocene	318 Teeth	2.20%	Compiled by Towel et al.^5^
	*Australopithecus sediba*	Early Pleistocene	16 Teeth	0.00%	Compiled by Towel et al.^5^
	*Homo naledi*	Mid-Pleistocene	147 Teeth	1.40%	Compiled by Towel et al.^5^
Artiodactyla	*Tethytragus sp.*	Mid-Miocene	1 Individual	–	Sala-Burgos et al.^33^
Carnivora	*Protarctos abstrusus*	Mid-Pliocene	2 Individuals	100%	Wang et al.^57^
	*Arctodus simus*	Late Pleistocene	33 Individuals	15.15%	Figueirido et al.^9^

Here, we present the first description of dental caries in a sample of an Early Eocene (~ 54 million years ago) mammal, the stem primate *Microsyops latidens*. This sample represents the earliest known incidences of caries among fossil mammals and the largest known collection of carious individuals for any fossil vertebrate taxon. These specimens were collected over the course of an almost fifty-year field project co-lead by Drs. Kenneth D. Rose, Thomas M. Bown, and (more recently) Amy E. Chew from the Willwood Formation of the southern Bighorn Basin of Wyoming, USA, an area that has produced the largest sample of stratigraphically controlled mammalian specimens in the world^34^.

First, we visually identify dental caries in our sample (n = 1030). We examine how closely dental pathology and functional dental form in *M. latidens* are associated with each other through time using previously collected dental topographic data from Selig et al.^35^, as a means for reconstructing dietary change in *M. latidens* and consider the degree to which dental caries serves as an additional dietary proxy. We also provide diagnostic criteria for caries in fossil mammal teeth. It should be noted that methods such as DTA pick up functional shape that has arisen out of an animal’s adaptation to their diet. This means that when examining change through time, dental topography changes at a slow pace over several generations. Methods such as microwear analysis (which measure scratches and pits on the teeth that can be correlated to patterns of diet) operate on a much shorter timescale, where the last few meals an animal eats are recorded in the microwear. Should dental caries serve as a proxy for examining dietary change through time, it too would operate at a faster time scale (though not as fast as microwear), as increased sugars in the diet would start to cause caries almost immediately. In this sense, analysis of dental caries would fill an intermediate gap in the timescale that can be addressed.

## Materials

### The study species

Plesiadapiforms were a paraphyletic group consisting of 11 families^36,37^ commonly regarded as stem primates^38^, meaning they have no living descendants. Microsyopidae^39^ is a family of plesiadapiform frequently recovered from the Bighorn Basin of Wyoming and are easily identified by their spearhead-shaped lower central incisors^37,40–42^. In phylogenetic analyses, the family is often found to have been among the most basal of primates^38,43^, though this classification is based mainly on dental characters; Microsyopidae sits outside of Primates (but still very closely related) when cranial characters are considered alone^44–46^. Although there is debate about the phylogenetic position of microsyopids and their relationship to primates, this argument ultimately does not change the nature of the current analysis.

Microsyopids were the second longest lived family of plesiadapiform, persisting from the Late Paleocene to the Late Eocene, a period of over 20 million years, with most species known from Western North America^37^. *Microsyops latidens*, known from the Early Eocene (~ 54 million years ago), is one of the best-known microsyopid species, represented by thousands of specimens^41^. The species likely weighed about 670 g on average based on body mass estimates derived from lower molar size^41^. Although represented by a large sample of dental remains, very little is known about the microsyopid skeleton^37^. However, they were likely arboreal, sharing similarities with arboreal primates and colugos^37^. In terms of their diet, *M. latidens* has been reconstructed as a likely omnivore, relying on a combination of fruits and possibly leaves based on previous dental topographic analysis and comparison to extant euarchontan taxa such as lorises, galagoes, treeshrews, and colugos^35^.

### The sample

Our sample consists of 1030 individuals, counted as the minimum number of individuals (MNI)^47^ at each meter level. Typically used in zooarchaeological analyses, the MNI is an estimate of the lowest possible number of individuals represented in a sample. It is calculated by dividing the number of dental elements into right and left and using the most abundant number of any one tooth position as the final estimate^47,48^. Although there are other methods for estimating sample size, such as counting the number of individual specimens, the MNI is more conservative as it avoids counting multiple elements that could have come from the same individual. Some have argued that the fraction of observable teeth with caries should be counted^49^. While this method provides a more accurate representation of the frequency of caries in a sample of loose teeth, teeth within jaws will be counted as independent units. Almost the entire sample of *M. latidens* is made up of teeth within dentaries, with very few isolated teeth. Moreover, extant primate samples have been counted using the number of individuals with caries, so using the MNI is a more appropriate count for the purposes of our comparative analysis.

Our sample spans about 544,000 years of the Early Eocene of the southern Bighorn Basin (between 425 and 600 m form the bottom of the stratigraphic section) based on the temporal values developed by Bown and Kraus^50^, the distribution of specimens in the section^34,51^, and the age of the top of the Willwood Formation stratigraphic section dated to 52.6 million years ago via ^40^Ar–^39^Ar dating^52^. As part of the previous dental topographic analysis, Selig et al.^35^ included 51 specimens with a lightly worn or unworn lower second molar (M_2_) from the same sample of *M. latidens*.

## Methods

### Identification of caries

A visual test is a common means for examining the presence of caries, with discoloration as a frequent cue in modern humans and animals, that also provides the basis for caries diagnosis in some recent fossil hominins e.g.,^5,6,53–55^. Due to the diagenetic nature of caries, there is typically a change in density associated with lesion formation, and a loss of density in the carious lesion itself, largely due to the demineralization of the hard tissues. Therefore, caries in recent and modern teeth can be diagnosed by touch using a dental probe^56^. Examining changes in density using micro-computed tomography (µCT) or x-ray techniques has also been used to diagnose caries in some fossil hominoids^5,6,10,30^, and Mid-Pliocene^57^ and Late Pleistocene bears^9^.

**Figure 1:**
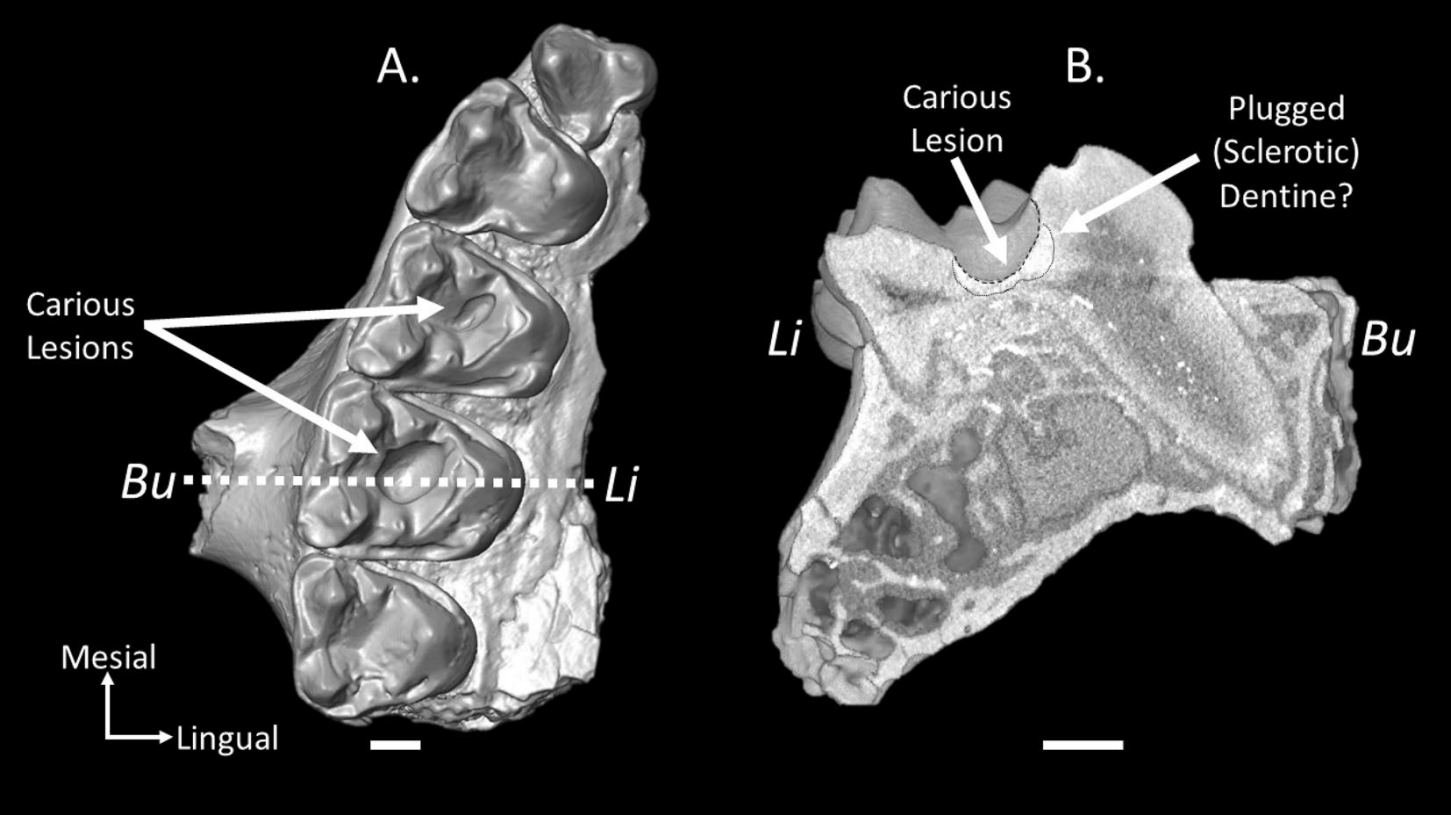
Micro-CT reconstruction of (**A**) the right upper jaw fragment (P^3^–M^3^) of *M. latidens* (USGS 17748) with carious lesions on the first and second molars and (**B**) a reconstruction of a slice through the caries in the second molar showing the internal morphology of the carious lesion. The slice is demarcated by the dashed line. Note that the enamel-dentine junction cannot be traced in this specimen. Identification of sclerotic dentine represents a hypothesis based on the observation of an area of higher density in the CT data at a depth that would be expected (based on the thin enamel of this taxon) to correspond to dentine. See Fuss et al.^10^ for another example of sclerotic dentine in a fossil primate specimen. *Bu* = buccal aspect, *Li* = lingual aspect. Scale bar = 1 mm.

**Figure 2:**
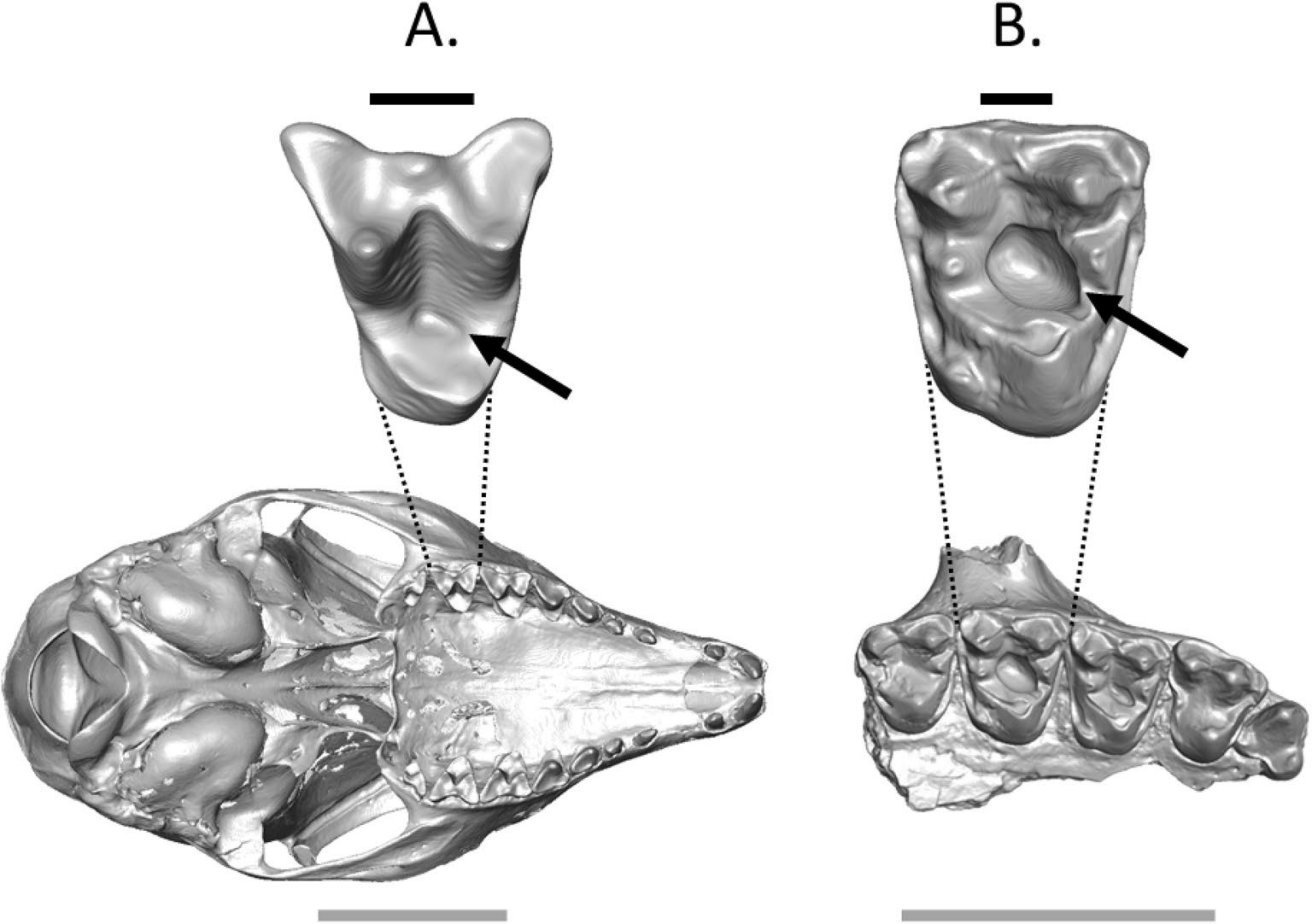
Micro-CT reconstruction of (**A**) the cranium of the extant treeshrew *Tupaia gracilis* (AMNH 103620) and (**B**) the right upper jaw fragment (P^3^–M^3^) of *M. latidens* (USGS 17748) with carious lesions. Enlarged second molars, with black arrows demarcating the carious lesions, demonstrate that the lesions are positioned in the same location in both the extant and extinct taxa. This is likely due to the fact that the tongue has a difficult time reaching the occlusal basin to clean the tooth, leading to accelerated carious activity and decay in this location. Grey scale bars = 1 cm, black scale bars = 1 mm.

The density of enamel and dentine in older specimens changes due to taphonomic processes (the process of fossilization), as does the color of the enamel and dentine, meaning that other methods are necessary to identify carious lesions in much older specimens. For example, lesions in the included sample were not necessarily darker or lighter in color than the enamel or dentine of the same tooth, because the teeth were typically dark brown or black overall, as is common for fossils from the Bighorn Basin. Therefore, discoloration could not be used as a criterion to identify caries in our sample. We used the following features to diagnose the presence of caries in our sample, and propose that these methods can be applied to the analysis of caries in other fossil specimens (see Fig. 1):

1. Smooth, rounded lesion: the shape of the lesion is smooth and rounded. As the lesion spreads through the enamel and into the dentine, it begins to inflate inwards as it reaches the softer dentine. Taphonomic processes, such as breakage, would be expected to leave more jagged edges around breaks or cracks, and would not necessarily be expected to produce a lesion that is wider internally than at the surface.
2. Presence of plugged (sclerotic) dentine: as dentine is exposed by caries or wear, mineralized material fills the hollow dentine tubules to prevent further destruction of the dentine and to prevent microorganisms from entering the pulp during life. This increases the radio-density of the dentine around the carious lesion, which is brighter in a µCT scan^10^. However, depending on the diagenesis of the tooth, this feature may or may not be observable.
3. Presence of caries in ‘hard to reach’ places: the tongue cannot easily reach areas such as the occlusal basin of the molars meaning food is more likely to become trapped and remain in place within the occlusal basin. This has been argued as a cause of high caries prevalence in some living primates such as *Cebus capucinus*^58^ and may be a common factor causing caries in other animals (such as treeshrews, as observed by the author) that are characterized by molars with tall cusps, as is the case with *M. latidens* (Fig. 2). We should note that while this particular type of dental morphology could lead to caries formation, it could also lead to other dental pathologies such as erosion from acid wear if acidic foods were to become trapped in the occlusal basins for long periods of time (but see below).

### Dental topographic analysis

Selig et al.^35^ previously quantified the dental topography of *M. latidens* using three metrics: Dirichlet normal energy (DNE), which is a measure of surface curvature, three-dimensional orientation patch count rotated (3D-OPCR), which is a measure of complexity, and the relief index (RFI), which is a measure of crown relief. As discussed earlier, all three of these metrics have been used to study variation in dental form among living mammals, and all have been shown to covary with diet e.g.,^16,24–26,59,60^. For example, mammals that consume foods that are hard to process orally, such as structurally complex plant parts or insect exoskeletons, typically have molars that are more highly curved and crested, more complex, and that are taller, compared to mammals that eat soft diets, such as fruits. Therefore, insectivores and folivores typically have molars with higher topographic values compared to the flat and simple teeth of fruit eating mammals. We can therefore use dental topographic signals to study not only what diet an extinct mammal may have been eating, but we can also trace how their dental form (and therefore their diet) changed over time.

To measure the dental topography, Selig et al.^35^ µCT scanned jaws and individual teeth, converted those µCT scans into computerized models or meshes, and prepped them using conventional methodology for the analysis of dental topography, with meshes simplified to 10,000 faces and smoothed 100 times^16–18,24,60–62^. See Selig et al.^35^ for more details on mesh preparation and measurement of the topographic metrics.

### Statistical analysis

To test if caries presence in *M. latidens* is more frequent than we might expect, we ran a chi-squared test on the association between caries presence and absence compared to samples of previously studied living primate taxa (see Table S1 for list of taxa). We also use the chi-squared test to assess whether or not there was an association between caries presence/absence based on binned meter levels to examine if there are any particular points in time where caries frequency is higher in *M. latidens* than might be expected. We used an alpha value of 0.05 and calculated the adjusted residuals with a 95% confidence interval of ± 1.96. The chi-squared tests were performed using PAST 4.02^63^.

## Results

**Figure 3:**
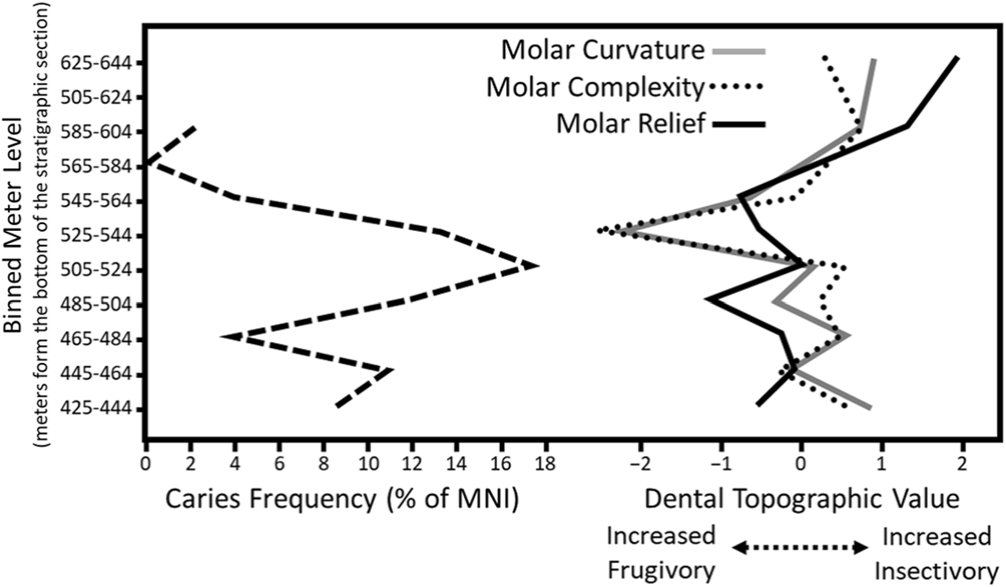
Results of the measurement of caries frequency over time and Z-score transformed results of the dental topographic analysis from Selig et al.^35^. Results are presented as mean values from each 20-m interval (or bin) form the bottom of the stratigraphic section. Note that during the period when caries frequency is highest (17.24% MNI from 505 to 524 m from the bottom of the stratigraphic section), molar curvature and molar complexity decrease (although slightly later from 525 to 544 m from the bottom of the section) suggesting that both dental topography and caries frequency are capturing a similar shift in diet during this period in time.

**Figure 4:**
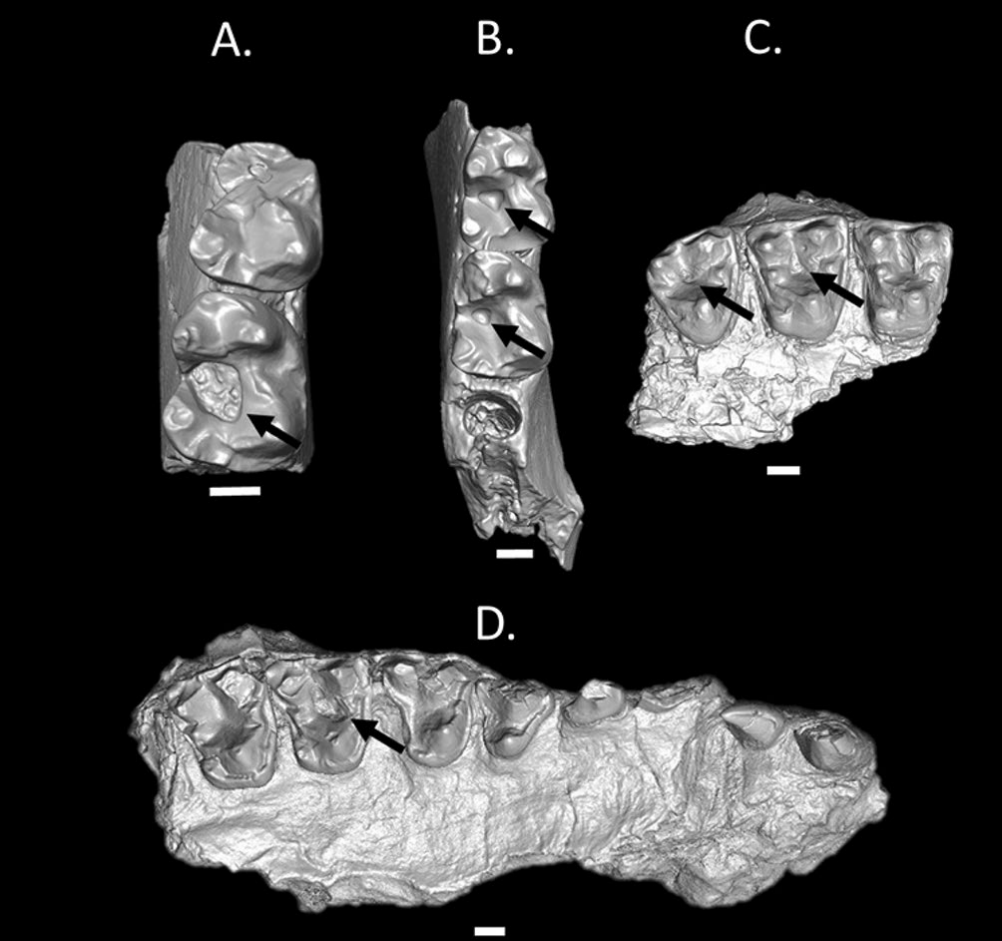
Micro-CT reconstructions of (**A**) a lower right jaw fragment (M_1_–M_2_) (USGS 3787), (**B**) a lower right jaw fragment (M_1_–M_2_) (USNM 537939), (**C**) an upper right jaw (M^1^–M^3^) (USGS 1109), and (**D**) an upper right jaw (I^1^–M^2^) (USGS 9194). Note that in each tooth, the caries is primary and located in the occlusal basin. Black arrows denote the carious lesions. Scale bar = 1 mm. Buccal is towards the right in the lower teeth and towards the top in the upper teeth.

In our sample of 1030 individuals, we identified caries in 77 of those individuals, which is 7.48% of the sample (Tables S2, S3). This is comparable to studies^2,10^ detailing modern primate species such as *Pan* (8.00%) and *Saguinus* (9.30%). Results of the chi-squared test (Tables S4, S5) suggest that there is a significant association between caries presence and absence among *M. latidens* and extant taxa (*χ*^2^ = 997.94, *P* = 5.37E−197). The adjusted residuals suggest that only *Cebus* and *Saguinus* are characterized by a higher presence of caries than expected compared to *M. latidens* (Table S5)*. Microsyops latidens* has far more caries than would be expected based on the rest of the extant taxa included in the analysis. When specimens are binned at 20-m intervals (Fig. 3), caries frequencies peak as high as 17.24% of the MNI (in the bin from 505 to 524 m from the bottom of the stratigraphic section). Results of the chi-squared test (Tables S6, S7) of caries presence/absence by meter level are also significant (*χ*^2^ = 24.387, *P* = 0.0009741). The adjusted residuals suggest that meter level intervals of 485–504 m and 505–524 m are characterized by significantly more caries than would be expected, whereas the intervals of 465–484 m and 545–564 m are characterized by significantly fewer caries (Table S7). All caries identified in our sample are primary occlusal caries located in the occlusal basin (Fig. 4). Carious lesions were only identified on the postcanine teeth, though this may be due to the fact that the anterior dentition is less frequently recovered in the fossil record.

In their analysis of *M. latidens* dental topography, Selig et al.^35^ suggested that the diet of *M. latidens* seems to remain relatively constant during the period from which the species is known. However, they noted that there is a single M_2_ specimen (in the bin from 525 to 544 m from the bottom of the section) that exhibits lower topographic values compared to most other specimens in their sample. They could not determine if this represented an actual shift in diet due to the small sample size but noted that there were 3 lower fourth premolars from the same time period that also exhibited lower topographic values. Selig et al.^35^ therefore suggested that this could indicate a shift towards greater frugivory around 525–544 m from the bottom of the section. Our current analysis would be consistent with this finding, with caries frequency peaking at 17.24% in the preceding bin (505–524 m from the bottom of the section, n = 5 with caries), and at 12.50% in the same bin as the peak identified by Selig et al.^35^ (525–544 m from the bottom of the section, n = 8 with caries).

## Discussion

Our results suggest that caries frequency was high at times in the past among *Microsyops latidens*, with large proportions of individuals afflicted by the disease in particular intervals of time (Fig. 3). With 7.48% of individuals showing caries overall, this is higher than the frequency observed in several living primate species, including highly frugivorous spider monkeys (*Ateles* sp.), woolly monkeys (*Lagothrix* sp.)*,* and titi monkeys (*Callicebus* sp.)^2^ (Table S1). In our sample, we identified only primary occlusal caries and no incidents of secondary or interproximal caries. This contrasts with the results from other studies suggesting that primary caries is rare in the wild^10,64^. Fuss et al.^10^, for example, found that only a small percent of caries in wild chimpanzees were primary (5/2890 [0.17%] of teeth analyzed). Indeed, among wild primates, secondary occlusal caries is most common, generally initiated by dental wear or fractures^10,64^. Our results are most consistent with the frequencies observed in wild tamarins (*Saguinus* sp.) (Table S1), which are known to consume high levels of sugar-rich foods like fruit and sap^65^. However, caries frequencies were at times as high as those observed in wild capuchins (*Cebus* sp.) (Table S1). Smith et al.^58^ noted caries in 26% of teeth studied in a sample of wild white-faced capuchin (*Cebus capucinus*) and attributed this high frequency to occlusal surface morphology. They argued that food was more likely to become trapped in the occlusal basins of capuchins compared to spider monkeys or howler monkeys (*Alouatta*), leading to a higher prevalence of caries in capuchins. Although this does not explain the changing frequency over time of caries observed in *M. latidens*, it is consistent with where on the tooth carious lesions appeared in this species.

Acidic foods becoming trapped in the occlusal basin could also lead to the formation of deep, pit-like features that are not caries. However, severe acid erosion is quite rare in non-humans. Dumont^71^ suggested that frugivorous mammals likely have high salivary pH to buffer against the erosive effects of an acidic diet. Subsequently, Cuozzo et al.^72^ found that ring-tailed lemurs (*Lemur catta*) have high salivary pH. They note that alkaline saliva likely limits the erosive effects of acid on the teeth, in light of the fact that some populations of *L. catta* consume highly acidic tamarind pods. If the features we observed in *M. latidens* were due to the processes of acid erosion as opposed to carious activity, we would expect the lesions would not be as severe as they are in many of our specimens given the buffering properties of saliva.

In their analysis of *M. latidens* dental topography, Selig et al.^35^ examined intraspecific variation in molar shape through time. Because of the low degree of variation they observed, they argued that the diet of *M. latidens* likely did not undergo significant change through time. They noted only a single period (525–544 m from the bottom of the section) when diet may have fluctuated, with a dental topographic signal consistent with increased frugivory. As discussed above, we note increased caries frequency at a similar point in time in the present analysis. We do note a gap in time between when we see a shift in caries frequency and when we see a shift in dental topography, with the signals from the caries record appearing earlier. This is not surprising as it is likely that caries frequency would change almost immediately following an increased consumption of sugars, whereas an adaptive change in dental morphology requires several generations. Overall, our results mean that two proxies for diet (dental topography and caries frequency) are suggestive of a transition to higher frugivory during a particular period of time (or at least to foods that contain more sugars) in *M. latidens*, meaning this may represent evidence for a meaningful shift in diet. This shift in diet may have lasted on the order of 10,000–20,000 years before returning to the baseline diet with less fruit/ sugars but does seem to represent a meaningful change in diet in this species.

Dietary shifts are important ecological events affecting the biology of extinct animals. These events provide information for understanding how and why species immigrate, emigrate, go extinct, compete with others, or adapt and evolve. Shifts in diet have been observed in other plesiadapiform lineages^21,66^, however, less is known about dietary shifts within single plesiadapiform species (but see Selig et al.^35,42^). Study of caries frequencies through time provides another tool for palaeontologists to reconstruct patterns of diet in extinct animals. Using this methodology, our results suggest that *M. latidens* underwent a dietary change resulting in an adaptive shift in the form of their teeth, and in the prevalence of dental disease. Although it is difficult to know exactly why such a shift occurred, it is well established that the Early Eocene was characterized by periods of climatic flux, with local temperatures increasing and decreasing through time^51,67–69^. Although palaeoclimatic data are currently lacking for the period and region from which *M. latidens* is known, previous study of dental form in earlier species of microsyopids from the same region demonstrated that local climate change may have been a factor leading to dietary change in this group^42^. *Microsyops latidens* may have relied on food sources that were higher in sugar, and therefore more cariogenic, during periods of climatic flux as a result of increased competition for limited food sources, or a change in the food sources that were available. This, in turn, may have led to the noted increase in caries frequency. As more palaeoclimatic data become available, it is possible that we will see evidence of further climatic change during this period, which may have affected the food sources available to *M. latidens.*

Our study provides the earliest collection of dental caries in a fossil vertebrate species, and the first analysis of dental caries frequency through time in any fossil taxon. Our study, therefore, provides a methodology and framework for examining caries frequency in deep time. Due to the close relationship between caries frequency and the results of the dental topographic analysis (a known method for studying diet), we suggest that the examination of caries frequency is a valid methodology for examining dietary change through time, which may operate on a more immediate timescale, and so provide a relevant alternative to DTA, which requires evolutionary shifts in morphology.

## Supplementary Information

Supplementary Table S1.

Supplementary Table S2.

Supplementary Table S3.

Supplementary Table S4.

Supplementary Table S5.

Supplementary Table S6.

Supplementary Table S7.
