# Supplementary material for: The largest and earliest known sample of dental caries in an extinct mammal (Mammalia, Euarchonta, Microsyops latidens) and its ecological implications
Source: Sci Rep. 2021 Sep 9;11:15920. doi: 10.1038/s41598-021-95330-x (PMC8429469; doi:10.1038/s41598-021-95330-x)
Supplement: Supplementary file 1 — Supplementary Table S1. [file 41598_2021_95330_MOESM1_ESM.docx]

| Taxon | Diet | Specimens Studied | % Caries |
| --- | --- | --- | --- |
| *Cebus sp.* | Omnivorous | 153 Individuals | 26.80% |
| *Saimiri sp.* | Omnivorous | 201 Individuals | 17.90% |
| *Saguinus sp.* | Frugivorous | 86 Individuals | 9.30% |
| *Pan sp.* | Omnivorous | 725 Individuals | 8.00% |
| *Pongo sp.* | Frugivorous | 513 Individuals | 5.80% |
| *Papio sp.* | Omnivorous | 74 Individuals | 5.40% |
| *Ateles sp.* | Frugivorous | 381 Individuals | 4.70% |
| *Lagothrix sp.* | Frugivorous | 82 Individuals | 1.20% |
| *Macaca sp.* | Omnivorous | 773 Individuals | 1.00% |
| *Callicebus sp.* | Omnivorous | 122 Individuals | 0.80% |
| *Pithecia sp.* | Frugivorous | 148 Individuals | 0.70% |
| *Cercopithecus sp.* | Omnivorous | 3,436 Individuals | 0.61% |
| *Hylobates sp.* | Frugivorous | 1,427 Individuals | 0.40% |
| *Presbytis sp.* | Omnivorous | 272 Individuals | 0.40% |
| *Gorilla sp.* | Folivorous | 1,323 Individuals | 0.30% |
| *Cercocebus sp.* | Omnivorous | 376 Individuals | 0.30% |
| *Alouatta sp.* | Folivorous | 1,173 Individuals | 0.30% |
| *Erythrocebus sp.* | Omnivorous | 42 Individuals | 0.00% |
| *Mandrillus sp.* | Omnivorous | 36 Individuals | 0.00% |
| *Theropithecus gelada* | Graminivorous | 21 Individuals | 0.00% |
| *Colobus sp.* | Folivorous | 59 Individuals | 0.00% |
| *Nasalis sp.* | Folivorous | 67 Individuals | 0.00% |
